# Motorcycle-related head and neck injuries: increased risk among ethnic minorities

**DOI:** 10.1186/s13584-020-00428-8

**Published:** 2020-12-08

**Authors:** Irit Cohen-Manheim, Irina Radomislensky, Maya Siman-Tov, Israel Trauma Group, A. Acker, N. Aviran, H. Bahouth, A. Bar, A. Becker, M. Ben Ely, D. Fadeev, I. Grevtsev, I. Jeroukhimov, A. Kedar, A. Korin, A. Lerner, M. Qarawany, A. D. Schwarz, W. Shomar, D. Soffer, M. Stein, M. Venturero, M. Weiss, O. Yaslowitz, I. Zoarets, Kobi Peleg

**Affiliations:** 1 Israel National Center for Trauma & Emergency Medicine Research, The Gertner Institute for Epidemiology and Health Policy Research, Sheba Medical Center, Tel-Hashomer, Ramat-Gan, 5265601 Israel; 2 The Department of Emergency Management & Disaster Medicine, School of Public Health, Sackler Faculty of Medicine, Tel-Aviv University, Tel Aviv-Yafo, Israel

**Keywords:** Head and neck injury, Traumatic brain injury, Injury prevention, Ethnicity, Jews, Arabs, Motorcycle, Helmet

## Abstract

**Background:**

Ethnic disparities have been associated with injury and mortality. The impact of ethnicity on head and neck injury (HNI), traumatic brain injury (TBI), in-hospital mortality and resource utilization following a motorcycle crash (MCC) is undetermined. This study explored the influence of ethnicity in these aspects and the effect of helmet use on HNI and TBI following a MCC.

**Methods:**

The National Trauma Registry provided hospitalization data on motorcycle riders and passengers between 2008 and 2017. Ethnicity was classified as Jews or Arabs, the two major ethnic groups in Israel. Univariate followed by multivariable logistic models were applied to examine ethnic disparities. Mediation effect was tested by structural equation modeling.

**Results:**

Among 6073 MCC casualties, Arabs had increased odds of HNI (OR = 1.37,95% CI = 1.12–1.65) and TBI (OR = 1.51,95%CI = 1.12–1.99), and a six-fold decreased odds of helmet use (OR = 0.16,95%CI = 0.12–0.22). The HNI and TBI associations with ethnicity were mediated by helmet use. Arabs had significantly higher odds for admission to intensive care unit (OR = 1.36,95%CI = 1.00–1.83), and lower odds for ambulance evacuation (OR = 0.73,95%CI = 0.61–0.89) and discharge to rehabilitation (OR = 0.55,95%CI = 0.39–0.7). In-hospital mortality was not associated with ethnicity.

**Conclusions:**

Helmet non-use is an important etiologic factor associated with motorcycle-related HNI and TBI among Arabs. While in Israel, ethnic equality exists in in-hospital health care, disparities in ambulance and rehabilitation utilization was found. Intervention programs should target the Arab population and focus on helmet compliance.

## Introduction

Motorcycle crashes (MCCs) are a significant cause of traumatic injury and mortality, resulting in approximately 88,000 casualties and 5029 deaths in the United States in 2015. Per vehicle mile travelled, motorcyclist fatalities were nearly 29-fold in comparison to car occupant fatalities and motorcyclists were five times more likely to be injured [1].

Head injury is most common among fatally injured motorcyclists [2]. Helmet use has been reported to reduce fatalities by 37% [3] and brain injuries by 67% [4], as well as to reduce hospitalization costs [5].

Ethnic disparities associated with traumatic injuries [6–10] and health care resource utilization [11] are well documented. In general, ethnic minorities are at greater risk of injury [6–9, 12, 13] and mortality [10, 12, 14, 15], along with higher resource utilization [11] than their ethnic majority counterparts. However, the evidence of ethnic disparities specific to MCC-related injury [8] and mortality [14] is sparse and inconclusive. In the Netherlands [11], motorcycle fatalities were significantly lower among ethnic minorities compared with native Dutch, while in the US [12], in comparison to non-Hispanic Whites, ethnic minorities were characterized with excess motor vehicle crash fatality risk. We are unaware of reports describing ethnic inequalities specific to MCC-related head and neck injuries (HNIs), traumatic brain injury (TBI) and hospital resource utilization.

In Israel, motorcycles are a popular mode of transportation; the number of registered motorcycles has nearly doubled from 77,472 in 2000 to 130,442 in 2016 [13]. Whereas motorcycles constitute 4% of total 3,239,305 registered vehicles and only 1.6% (928 million kilometers) of total vehicle kilometer travelled in 2016 [13], motorcycle injuries account for nearly 9% of road traffic motor collision injuries and motorcycle fatalities accounted for 12% of fatalities from motor collisions. These figures are translated to 1988 motorcycles involved in road accidents with causalities, 1905 injured motorcyclists, and 42 deaths in 2016. The corresponding numbers for 2017 were even higher: 2331 accidents, 2225 casualties, and 58 deaths [14, 15].

The population of Israel is comprised of two major ethnic groups: the Jewish majority (74.8% in 2016) and Arab minority (20.8%, i.e., Muslims, Circassians, Arab Christians, including Armenian, Druze and Lebanese). Ethnic groups differ in language, religion, culture, socioeconomic status, education and health-related characteristics. In 2014, 70.9% of Jewish 17 year olds were entitled to a high school diploma, compared with 45.9% of Arabs [16]. Life expectancy among Jewish men is 81.5 years and 84.7 years for women compared with 77.2 and 81.4 among Arabs, respectively [17]. In addition, in 2010, Arabs compared with Jews, had higher rates of traffic law violations and lower use of car seat restraints [18]. Interestingly, motorcycle related injuries were five-times greater among Israeli Jewish children compared with minorities [19].

The objectives of this study were to investigate HNI, TBI, in-hospital mortality and resource utilization between ethnic groups in Israel following a MCC and determine the contribution of helmet use. Decision makers should use this evidence-based nationwide data to develop and implement targeted interventions.

## Methods

This was a cohort study based on the Israeli National Trauma Registry (INTR) between 2008 and 2017. The study included MCC-hospitalized casualties with an ICD-9-CM external cause of injury code (E-code) for motorcycle riders (E810-E825) and passengers (E810-E825), with the digit 2 or 3 after the decimal point, respectively, for a traffic or non-traffic accident.

All trauma patients admitted to the department of emergency medicine (ER) and hospitalized, died in the ER, or transferred to or from another hospital are included in the INTR. The registry excludes casualties who died at the scene or on the way to the hospital and admissions 72 h or more following the event.

Trauma registrars record the data at each hospital, followed by quality assurance. Demographic characteristics included age, gender and ethnicity (Jews and Arabs). Crash characteristics comprised motorcyclist position (rider or passenger), collision type, injury location, helmet use, and hospital admission time. Injury characteristics included HNI, TBI, other injured body region and Injury Severity Score (ISS). The Abbreviated Injury Scale (AIS) codes and scores of the nine body regions were used for recognizing the injured body regions. Severe injury was defined as AIS ≥ 3. Injury severity of multiple injuries was based on ISS, calculated by summing the squares of the severity digit in the AIS [20] of the most severe injuries in up to three of six predefined body regions, categorized as 1–8 (minor), 9–14 (moderate), 16–24 (severe) and 25–75 (critical) [21, 22]. TBI was defined as any recorded evidence of intracranial injury in accordance with the AIS 1990 Revision Manual [23]. Resource utilization was assessed by evacuation mode, intensive care unit (ICU) admissions (0 / ≥1 days), undergone surgery (yes/no), length of stay (LOS, ≤7/ > 7 days), discharged to rehabilitation (yes/no) and in-hospital mortality.

**Fig. 1:**
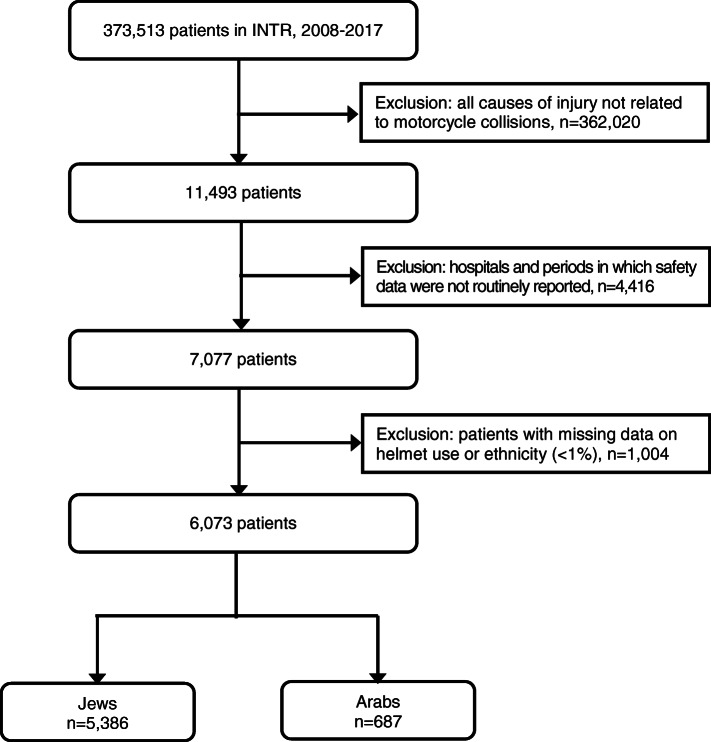
Consort diagram illustrating selection of patients in the INTR

This study was restricted to hospitals and periods in which the use of safety devices was routinely reported, i.e., the missing data regarding safety did not exceed 35%. Missing data on helmet use and unknown ethnicity were excluded from the analyses (Fig. 1).

The study was approved by the Sheba Medical Centre Helsinki Committee (SMC-5138-18).

### Statistical analysis

Data were analyzed by Chi-squared (χ2) test. Univariate analyses and multiple logistic models were applied to examine the associations of ethnicity with HNI, TBI, in-hospital mortality and hospital resource utilization. Multivariable models were adjusted for demographic variables (age and gender) and motorcyclist position, the latter reported to be associated with motorcyclist mortality [24] and trauma head injuries [25]. HNI and TBI models were further adjusted for helmet use to explore its contribution. Resource utilization models were also adjusted for ISS, as it was significantly associated with both ethnicity and resource utilization variables.

Mediation effect was tested by structural equation modeling (SEM) with a DWLS estimator, suited for binary variables, and bootstrapping 1000 samples. Effect modification of the ethnicity-HNI and TBI associations were tested for helmet use in separate regression models using multiplicative terms.

Jewish and Arab MCC incidence rates per 100,000 residents were calculated by dividing the number of hospitalizations due to MCC by the total population estimates. The average-year estimates of the population was sourced from the national Central Bureau of Statistics [26] for 2016. In order to account for the differences in the age structure of the ethnic populations being compared, the Jewish age-structure population in Israel as of 2016 was used as a standard in computing the age-standardized rate among Arabs.

Nominal two sided *p*-values are reported. The mediation analysis was executed using the ‘Lavaan’ package in R, version 3.4.1 [27]. The remaining statistical analyses were carried out using SAS software, version 9.2 (SAS Institute, Cary, NC, USA),

## Results

### Demographic characteristics

The study included 6073 MCC related hospitalized patients, 88.7% Jews and 11.3% Arabs (Table 1). The 2016 age-standardized rate among Arabs was 5.75/100,000 residents compared with the crude rate of 8.68/100,000 among Jews. The 16–25 age group (42.3%) and males (93.9%) were at greatest risk. Arabs, compared with Jews, were younger (64.9% vs. 39.5% were aged 16-25y, *p* < 0.001) and more likely to be male (98.4% vs. 93.3%, p < 0.001).

**Table 1:** Demographic, crash, injury and hospitalization characteristics of motorcycle crash-related injuries admitted to hospitals by ethnicity: 2008–2017

	Total	Jews	Arabs	***P*** value
**Demographic characteristics**				
**n (%)**	6073 (100.0)	5386 (88.7)	687 (11.3)	
**Age group (y)**^†^				
< 16	37 (0.6)	31 (0.6)	6 (0.9)	< 0.0001
16–25	2564 (42.3)	2122 (39.5)	442 (64.9)	
26–35	1519 (25.1)	1372 (25.5)	147 (21.6)	
36–45	943 (15.6)	888 (16.5)	55 (8.1)	
46–55	554 (9.1)	537 (10.0)	17 (2.5)	
56–65	318 (5.3)	307 (5.7)	11 (1.6)	
> 65	123 (2.0)	120 (2.2)	3 (0.4)	
**Male Gender**	5700 (93.9)	5024 (93.3)	676 (98.4)	< 0.0001
**Crash characteristics**				
**Motorcyclist position**				
Rider	5809 (95.7)	5163 (95.9)	646 (94.0)	0.027
Passenger	264 (4.4)	223 (4.1)	41 (6.0)	
**Type of collision**				
Motor vehicle	2900 (47.8)	2578 (47.9)	322 (46.9)	0.623
Self-accident	2761 (45.5)	2457 (45.6)	304 (44.3)	0.498
**Helmet use**	5844 (96.2)	5258 (97.6)	586 (85.3)	< 0.0001
**Location of Injury**				0.007
Interurban road	1669 (27.5)	1490 (27.7)	179 (26.1)	
Urban road	3550 (58.5)	3140 (58.3)	410 (59.7)	
Dirt road	136 (2.2)	109 (2.0)	27 (3.9)	
Unknown	718 (11.8)	647 (12.0)	71 (10.3)	
**Admission time**				
00:00–05:59	567 (9.3)	494 (9.2)	73 (10.6)	< 0.0001
06:00–11:59	1279 (21.1)	1180 (21.9)	99 (14.4)	
12:00–17:59	2322 (38.2)	2063 (38.3)	259 (37.7)	
18:00–23:59	1905 (31.4)	1649 (30.6)	256 (37.3)	
**Injury characteristics**				
**HNI and TBI**				
HNI	1117 (18.4)	950 (17.6)	167 (24.3)	< 0.0001
Severe HNI (AIS ≥ 3)	466 (7.7)	386 (7.2)	80 (11.6)	< 0.0001
TBI	399 (6.6)	334 (6.2)	65 (9.5)	0.001
Other Injured body region*				
Face	770 (12.7)	663 (12.3)	107 (15.6)	0.015
Upper extremities	2424 (39.9)	2127 (39.5)	297 (43.2)	0.059
Lower extremities	3592 (59.2)	3177 (59.0)	415 (60.4)	0.475
Abdomen	1213 (20.0)	1039 (19.3)	174 (25.3)	0.002
Thorax	1504 (24.8)	1307 (21.3)	197 (28.7)	0.012
Spine	565 (9.3)	484 (9.0)	81 (11.8)	0.017
External	331 (5.5)	308 (5.7)	23 (3.4)	0.009
**ISS**				0.006
Minor (ISS 1–8)	3242 (53.4)	2911 (54.1)	331 (48.2)	
Moderate (ISS 9–14)	1767 (29.1)	1560 (29.0)	207 (30.1)	
Severe (ISS 16–24)	517 (8.5)	448 (8.3)	69 (10.0)	
Critical (ISS 25–75)	547 (9.0)	467 (8.7)	80 (11.6)	
**Resource utilization**				
**Evacuation type**				
Ambulance	4702 (77.4)	4200 (78.0)	502 (73.1)	< 0.0001
Private car	916 (15.1)	806 (15.0)	110 (16.0)	
Helicopter	63 (1.0)	44 (0.82)	19 (2.8(	
Police/ other	18 (0.3)	11 (0.20)	7 (1.02)	
Unknown	374 (6.2)	325 (6.0)	49 (7.1)	
**Admitted to intensive care unit**	747 (12.3)	627 (11.6)	120 (17.5)	< 0.0001
**Undergone surgery**	2808 (46.2)	2494 (46.3)	314 (45.7)	0.767
**In-hospital length of stay (days) > 7**	1630 (26.8)	1421 (26.4)	209 (30.4)	0.025
**Discharged to rehabilitation**	540 (8.9)	493 (9.2)	47 (6.8)	0.045
**In-hospital mortality**	68 (1.1)	57 (1.1)	11 (1.6)	0.203

*Abbreviations*: *HNI* Head and neck injury, *TBI* Traumatic brain injury, *AIS* Abbreviated Injury Scale, *ISS* Injury Severity Score

*Numbers may not sum to total because of multiple regions

^†^Missing data: n=15

### Crash characteristics

The most common traffic crash was a collision with another motor vehicle (47.8%), equally distributed between ethnicities. Arabs, compared with Jews, were more likely to be involved in MCC from 18:00 to midnight (37.3% vs. 30.6%, *p* < 0.0001), to be a passenger (6.0% vs. 4.1%, *p* = 0.027) and ride on a dirt road (3.9% vs. 2.0%, *p* = 0.007) (Table 1).

### Helmet use

Arabs were less likely to use helmets (85.3% vs. 97.6%, *p* < 0.0001, Table 1) with 7-fold decreased odds of using one (OR = 0.14,95%CI = 0.11–0.19, *p* < 0.0001, not shown) compared to their Jewish counterparts. This association remained significant in a multivariable model adjusted for age, gender and motorcyclist position (rider vs. passenger) (OR = 0.16,95%CI = 0.12–0.22, *p* < 0.0001, not shown).

### HNI and TBI

The Arab minority sustained more HNI (24.3 vs. 17.6%, p < 0.0001), severe HNI (11.6% vs. 7.2%, p < 0.0001) and TBI (9.5% vs. 6 .2%, *p* = 0.0012, Table 1) compared with Jews. In multivariable models, controlling for age, gender and motorcyclist position, these associations remained significant (Table 2, Model II). Inclusion of helmet use in the regression models rendered the associations between ethnicity and HNI and TBI non-significant (Table 2, Model III), implying that helmet usage mediated these associations. Introducing a geographic measure (i.e., peripheral vs. central trauma center) into all models did not materially affect the association between ethnicity and HNI or TBI.

**Table 2:** Effect mediation models of ethnicity by helmet use on motorcycle crash-related HNI and TBI

	HNI	TBI
Unadjusted Model I		
Ethnicity (Arabs vs. Jews)	1.50 (1.24–1.80)^***^	1.57 (1.18–2.07)^***^
Adjusted Model II		
Ethnicity (Arabs vs. Jews)	1.37 (1.12–1.65)^**^	1.51 (1.12–1.99)^***^
Adjusted Model III		
Ethnicity (Arabs vs. Jews)	1.12 (0.91–1.37)	1.13 (0.82–1.53)

*HNI* Head and neck injury, *TBI* Traumatic brain injury

Numbers are odds ratios [ORs] and 95% confidence interval [CI]

Model I: Unadjusted

Model II: Adjusted for age, gender and motorcyclist position (rider/passenger)

Model III: Additionally adjusted for helmet use

^*^p < 0.05 ^**^p < 0.01^***^p < 0.001 ^****^p < 0.0001

**Table 3:** The association of ethnicity with motorcycle crash-related in-hospital mortality and hospital resource utilization

	In-hospital mortality	Ambulance evacuation	ICU admission	Hospital LOS > 7 days	Discharged to rehabilitation
Unadjusted Model I					
Ethnicity (Arabs vs. Jews)	1.53 (0.76–2.82)	0.78 (0.65–0.94) ^**^	1.61 (1.29–1.99)^****^	1.24 (1.04–1.47)*	0.74 (0.53–0.99)*
Adjusted Model II					
Ethnicity (Arabs vs. Jews)	1.21 (0.57–2.37)	0.73 (0.61–0.89)^**^	1.36 (1.00–1.83)*	1.10 (0.90–1.34)	0.55 (0.39–0.77)^***^

*ICU* Intensive care unit, *LOS* Length of stay, *ISS* Injury Severity Score

Numbers are odds ratios [ORs] and 95% confidence interval [CI]

Model I: Unadjusted

Model II: Adjusted for age, gender, motorcyclist position (rider/passenger) and ISS [1–8 (minor), 9–14 (moderate), 16–26 (severe), 25–75 (critical)]

^*^p ≤ 0.05 ^**^p < 0.01 ***p < 0.001 ****p < 0.0001

### Mediation effect

To test the mediation effect of ethnicity-HNI and ethnicity-TBI associations with helmet use (the mediator), the four steps by Baron and Kenny [28] were followed. Regression analyses were conducted, and the significance of the coefficients were examined for each path separately, as follows: (1) ethnicity affected the odds for HNI and TBI (Table 2, Model I), (2) ethnicity affected the likelihood to use a helmet (Table 1), and (3) helmet use negatively affected the likelihood of HNI (OR = 0.22,95% CI = 0.17–0.29, *p* < 0.0001) and TBI (OR = 0.19,95%CI = 0.14–0.27, p < 0.0001) (not shown). Step 4 included a multiple regression analysis with both ethnicity and helmet use. The results support mediation, as the effect of helmet use remained significant after controlling for ethnicity (all *p* < 0.001, not shown).

**Fig. 2:**
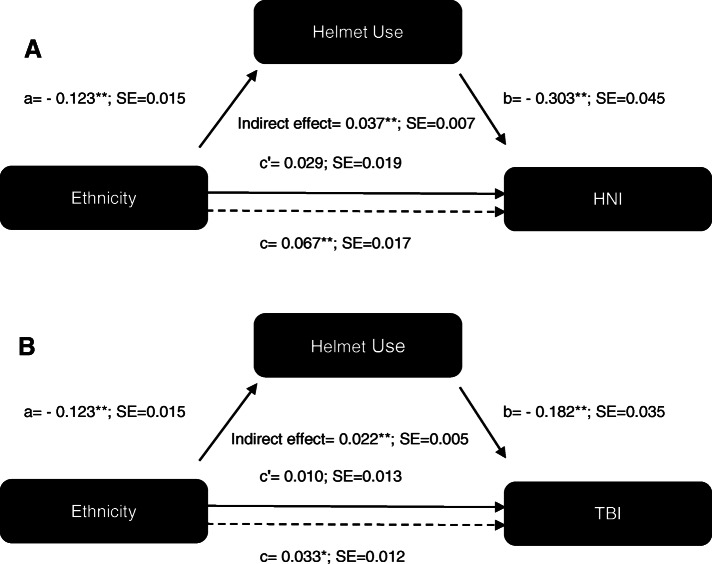
Full mediation models showing the effect of ethnicity on HNI (A) and TBI (B) through helmet use (the mediator)

The indirect pathway was calculated and tested for significance. As shown (Fig. 2), bootstrapping yielded a significant indirect effect (ab = 0.037 and 0.022 for models A and B, respectively, both *p* > 0.001) suggesting some form of mediation. The non-significant direct effect (c’), suggests full mediation. Sensitivity analysis adjusting for age, gender and motorcyclist position did not materially affect the results (not shown).

### Moderation effect

No evidence existed for effect modification of the ethnicity association with HNI and TBI in the multivariable models (p for interaction> 0.5 for all tested) (not shown).

### In-hospital mortality

In-hospital mortality following MCC was low for both ethnicities and non-significantly higher among Arabs in comparison with Jews (1.6% vs. 1.1%, *p* = 0.203, Table 1).

### Health resource utilization

Ambulance evacuation was higher among Jewish casualties (78.0% vs. 73.1%, *p* = 0.004, Table 1), whereas helicopter evacuation was greater among Arabs (2.8% vs. 0.8%, < 0.0001). This disparity was similar among severe/critical casualties evacuated by ambulance (87.5% vs. 74.5%, *p* < 0.0001) or helicopter (8.7% vs. 2.6%, *p* = 0.0002) (not shown). While ICU admission and LOS > 7 days were higher among Arabs (17.5% vs. 11.6%, p < 0.0001 and 30.4% vs. 26.4%, *p* = 0.025, respectively), discharge to a rehabilitation facility was greater among Jews (9.2% vs. 6.8%, *p* = 0.045) and there was no difference between Arabs and Jews being discharged to home (89.3 and 90.4%, respectively, not shown). In a multivariable analysis adjusting for age, gender, motorcyclist position and ISS, the difference between Arabs and Jews for hospital LOS > 7 days (Table 3, Model II) was no longer significant, whereas ambulance evacuation (OR = 0.73, 95%CI = 0.61–0.89, *p* = 0.002), ICU admission (OR = 1.36, 95%CI = 1.00–1.83) and discharge to rehabilitation (OR = 0.55, 95%CI = 0.39–0.77, *p* = 0.0006) remained significant. Repeating the analyses with further adjustment for a geographical measure (i.e., peripheral vs. central trauma center) did not materially affect the ethnicity-outcome associations. Subgroup analysis restricting the ICU admission analysis to severe HNIs, rendered the ethnicity-ICU admission non-significant, namely, ICU admission was higher among Arabs due to higher risk for severe HNI resulting from lower helmet use.

### Determinants of helmet non-use

**Fig. 3:**
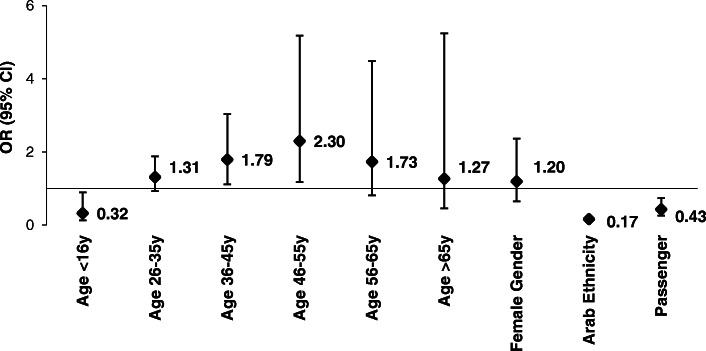
Adjusted odds ratios (ORs) for helmet use

A multivariate logistic model showed that children (< 16 year) were less likely to use helmets (OR = 0.32, 95%CI = 0.13–0.90, *p* = 0.020) compared to ages 16–25 (Fig. 3). Passengers, compared with riders, were less likely to use a helmet (OR = 0.43, 95%CI = 0.26–0.74, *p* = 0.001). Arabs, compared with Jews, were least likely to use a helmet (OR = 0.17, 95%CI = 0.12–0.22, *p* < 0.0001).

## Discussion

This study characterizes MCC-related HNI, TBI, in-hospital mortality and resource utilization among hospitalized Israeli Jews and Arabs. Due to lower helmet use, Arabs were more likely to endure HNI and TBI and to be admitted to the ICU following a MCC. Ethnicity may play a role in underutilization of ambulance and rehabilitation services. In-hospital mortality was not associated with ethnicity.

Higher risk for MCC injury per 100,000 Jewish residents corresponds with our previous analysis [19] reporting that Jewish children aged 0–17 sustained five times more MCC-related injuries than Arab children. The lower risk of injury among Arabs could be explained by their non-preference of this mode of transportation. According to the Central Bureau of Statistics (CBS), Jews comprise 89.2% of all motorcycle-licensed drivers compared with only 8.6% among Arabs, whereas the latter comprise 20.8% of the total population in Israel [29]. That is, the apparent discrepancy of greater proportion of motorcycle-related HNI and TBI versus the lower risk per 100,000 residents among Arabs could be explained by higher injury risk per kilometer travelled instead of a higher rate of exposure. We were unable to compute the injury risk per kilometer travelled or per registered vehicle, as such data by ethnicity are lacking. Higher risk per kilometer travelled could be related to various factors. For instance, younger age groups, which characterized Arab patients, are prone to more reckless, risky, and thrill-seeking behaviors [30]; however, current study incidence rates were age-adjusted and the ethnicity-injury associations persisted after adjusting for age. An alternative explanation may be related to newer and safer motorcycles and helmets, which are less common among Arabs, whom mostly live in low socioeconomic communities [31]. In contrast to the United States [7], which reported that ethnic minorities were characterized with an increased motor vehicle crash fatality risk attributed to, inter alia, higher rates of alcohol use, in Israel, riding under the influence of alcohol is rare among Arabs. According to the CBS, only 3 of 393 (0.8%) Arab motorcycle drivers involved in road accidents with casualties in 2018 were due to alcohol/drugs involvement, compared with 25 of 1801 (1.4%) Jewish drivers [32].

In view of the bulk literature that helmet use decreases severe head injuries and fatal injuries among motorcyclists [3, 4], this is the first study, to the best of our knowledge, to underline ethnic related modifiable risk factors associated with MCC-TBI and HNI, as evident by the mediation analysis reported herein.

In Israel, motorcyclist helmet use is mandatory and strictly enforced. Although the majority of Arab and Jewish patients wore a helmet, the usage was significantly lower among Arabs (85 and 98%, respectively). In addition to risky and illegal driving among Arabs reported by the Israeli National Road Safety Authority (NRSA) [18], as frequently found among ethnic minorities in other countries [7], our findings show lower compliance with helmet use which could be cultural-related. Fatalistic beliefs, which are greater among Arabs [33], could inhibit adoption of traffic laws and prevention behaviors. Perceived discrimination [34], as well as low socio-economic position, may be related to unsafe and unlawful driving culture among Arabs. Lastly, defiance of state authorities, as perceived by Arabs [34], may serve as a cause of unsafe driving and thus increase their susceptibility to lower compliance with helmet use.

Whereas helmet use explained the HNI and TBI ethnic differences, its non-use may also explain the higher rates of ICU admission among Arabs; indeed, comparing the ICU admission odds among severe head and neck injured motorcyclists, diminished the ethnic disparities.

We were unable to identify studies that have either examined ethnic disparities in HNI, TBI and resource utilization after a MCC, or that have examined helmet use as a mediator and/ or moderator in this context.

Health care access, rehabilitation and health insurance are important factors for injury outcome, and often lead to inequalities in medical care [14]. In Israel, all residents, regardless of ethnicity, religion and gender are provided with health-care services under the National Health Insurance Law [35]. Fees are non-conditional. Arabs and Jews are equally eligible to receive outpatient and inpatient health services and are treated in the same hospitals with the same health-care teams; therefore, health care inequalities are unlikely to play a part in explaining any ethnic differences. Our findings attest to the success of the Israeli healthcare and trauma system in delivering non-discriminating quality of care, regardless of ethnicity, as evident by the higher ICU admissions among the Arab minority and the non-differential rates of prolonged LOS (> 7 days) and in-hospital mortality.

Concordant to the observed underutilization of rehabilitation at discharge among Arabs, an earlier Israeli study found ethnicity, to be the salient barrier for rehabilitation, characterized with significantly lower rates among Arab patients [36]. The observed underutilization of both rehabilitation and ambulance service could be attributed to accessibility and geographic barriers. Studies have reported lower inpatient rehabilitation rates after stroke or hip fracture in the Northern and Jerusalem districts, which is attributed to a shortage in rehabilitation beds [37]. Likewise, 56 and 19% of Israeli Arabs live in the Northern district and Jerusalem, respectively, while only 11% live in the Central district; the corresponding numbers among Jews are 21, 12 and 52% [38]. While ambulance use may be related to availability in Arab communities [39], it is partially compensated by the significantly greater helicopter evacuations among Arabs, even after adjusting for ISS. Cultural attitudes may reflect the underutilization of rehabilitation services among Arabs. It is common for Arab families to reside one next to the other and be actively involved in each other’s lives [40].

The main strength of this study was the use of the National Trauma Registry, which provides current nation-wide data. Additionally, this study also found that ethnic disparities do exist following MCC and that these disparities are behavioral modifiable factors. Hence, building on our assessment, we propose that a multidimensional intervention be developed and should incorporate education, awareness, law enforcement, infrastructure, and community and religious respected individuals with an understanding of cultural attitudes and beliefs. Enforcing motorcycle helmet use and traffic laws, such as use of safety belts and child restraints, speeding, driving under the influence of alcohol and mobile phone use have had varying results to date [41]. Israel has recently developed at least two programs focusing on road safety behavior developed and customized to the needs of Israeli Arabs: (1) a specifically tailored-four and a half months-traffic enforcement program through an innovative 8 months public participation process. The experimental program involved key informants (local government leaders, local council officials, social workers, head of citizens’ groups, local religious leaders, school head-teachers, and police officers from the national transport police and the local police) which identified local road traffic problems and ‘dark’ hot spots (places where offenses and risky behavior recur but might not be known to the police) and accordingly planned related communication campaigns in selected minority communities [42]. (2) a nationwide program operated by the NRSA throughout the Arab society since 2017 [43]. The program is designed to guide, motivate and empower Arab road users to use a seat belt while focusing on improving the road safety climate, road safety behaviors and awareness. Following the implementation of intervention programs in Arab communities, a reduction in traffic violations (indicating an improvement in drivers’ behavior) was reported [42], as was a decrease in traffic related serious injury and mortality rate [43]. Although this is the lowest traffic casualties’ rate in the preceding 10 years, it is still not adequate and does not correspond to the proportion of Arabs in the general population [43]. Implementing an ongoing multi-strategic program that combines these efforts, including education, awareness, enforcement and participation of public and community key figures, which target all minority communities, is recommended. In addition to focusing on road safety behaviors, interventions should emphasize the safety value of helmet use and the consequences of non-use. Distribution of free or subsidized helmets to increase helmet accessibility [44] and using behavioral change principles through distribution of rewards or incentives [45] to reinforce the mandatory use of helmets should also be considered.

### Limitations

The present study has several limitations. First, this study includes only hospitalized patients, resulting in an underrepresentation of minor injuries and fatalities occurring at the scene of the event. Second, this study excluded hospitals and periods in which safety data were not routinely reported and patients with missing data regarding helmet use and ethnicity. Yet, this study covered five of all six Level I Trauma Centers in Israel and 11 out of 13 Level II Trauma Centers participating in the INTR. Furthermore, we compared the final patient cohort characteristics with all eligible patient characteristics to determine the extent to which the final cohort was representative of the total MCC population and found that the ethnic distribution was similar; the age, gender and ISS distributions were also similar between the groups being compared. Third, information on potential confounders as religious differences (Muslim vs. Christian vs. Druze), patient comorbidities, drugs, alcohol, vehicle speed and motorcycle and helmet model were unavailable to us and therefore were not adjusted for. Fourth, it was not possible to compute the injury risk per kilometer travelled or per registered vehicle, as such data by ethnicity are lacking.

## Conclusions

This study identified helmet use as a key factor in explaining the MCC-related ethnic disparity in HNI and TBI, and ethnicity, as a role player in out-of-hospital resource utilization (i.e., ambulance and rehabilitation services), but not in provision of in-hospital health care. Our results call for policy makers to allocate resources for sector-specific and cultural-oriented interventions to promote helmet use and improve enforcement actions. Other intervention strategies and resources may be necessary as well to mitigate ethnic disparities targeting minority groups, such as promoting the use of ambulance services and participation in rehabilitation programs. Finally, since reducing health related ethnic disparities is a global public health goal, research should continue to identify potential modifiable risk factors.

## Data Availability

The datasets generated and analyzed during the current study are not publicly available due to hospitalization privacy but are available from the corresponding author on reasonable request.

## Abbreviations

AIS: Abbreviated Injury Scale
HNI: Head and neck injury
ISS: Injury Severity Score
TBI: Traumatic brain injury
